# Sequential development of diffuse panbronchiolitis and myasthenia gravis after thymectomy for thymic neoplasm: a case report

**DOI:** 10.1186/s12890-024-03134-2

**Published:** 2024-07-02

**Authors:** Chun-Ying Chou, Min-Shu Hsieh, Ping-Hung Kuo

**Affiliations:** 1 Department of Internal Medicine, National Taiwan University Hospital, No. 7, Zhongshan S.Rd., Zhongzheng Dist. Taipei, 100 Taiwan; 2 Department of Pathology, National Taiwan University Hospital, No. 7, Zhongshan S.Rd., Zhongzheng Dist. Taipei, 100 Taiwan

**Keywords:** Diffuse panbronchiolitis, Myasthenia gravis, Thymoma, Thymic neoplasm, Case report

## Abstract

**Background:**

Myasthenia gravis (MG) is the most common paraneoplastic disorder associated with thymic neoplasms. MG can develop after thymectomy, and this condition is referred to post-thymectomy myasthenia gravis (PTMG). Diffuse panbronchiolitis (DPB), is a rare form of bronchiolitis and is largely restricted to East Asia, has been reported in association with thymic neoplasms. Only three cases of combined MG and DPB have been reported in the literature.

**Case presentation:**

A 45-year-old Taiwanese woman presented to our hospital with productive cough, rhinorrhea, anosmia, ear fullness, shortness of breath, and weight loss. She had a history of thymoma, and she underwent thymectomy with adjuvant radiotherapy 7 years ago. Chest computed tomography scan revealed diffuse bronchitis and bronchiolitis. DPB was confirmed after video-assisted thoracoscopic surgery lung biopsy, and repeated sputum cultures grew *Pseudomonas aeruginosa*. She has been on long-term oral azithromycin therapy thereafter. Intravenous antipseudomonal antibiotics, inhaled amikacin, as well as oral levofloxacin were administered. Three months after DPB diagnosis, she developed ptosis, muscle weakness, and hypercapnia requiring the use of noninvasive positive pressure ventilation. MG was diagnosed based on the acetylcholine receptor antibody and repetitive stimulation test results. Her muscle weakness gradually improved after pyridostigmine and corticosteroid therapies. Oral corticosteroids could be tapered off ten months after the diagnosis of MG. She is currently maintained on azithromycin, pyridostigmine, and inhaled amikacin therapies, with intravenous antibiotics administered occasionally during hospitalizations for respiratory infections.

**Conclusions:**

To our knowledge, this might be the first case report of sequential development of DPB followed by PTMG. The coexistence of these two disorders poses a therapeutic challenge for balancing infection control for DPB and immunosuppressant therapies for MG.

## Background

Thymomas and thymic carcinomas are rare primary tumors located in the mediastinum and derived from the thymic epithelium. Myasthenia gravis (MG) is the most prevalent type of paraneoplastic syndrome associated with these tumors [1]. Approximately 30% of patients with thymoma either present with or develop MG [2, 3]. Thymectomy should be performed as an oncological intervention if a thymoma is identified or strongly suspected to prevent local compression and possible spread to the thoracic cavity [4]. Nevertheless, after thymectomy, patients can develop post-thymectomy myasthenia gravis (PTMG). PTMG refers to the subsequent development of MG after radical surgical resection in patients with thymoma who did not exhibit any signs of MG before surgery. PTMG might be misdiagnosed not only because of its rare incidence, which has been reported to be between 0.97% and 13.39% in previous studies, but also due to the uncertain interval between the removal of thymoma and the new onset of PTMG, which can range from 3 days to over 14 years [5].

Bronchiectasis has been recognized as a potential complication associated with thymic neoplasms, and there have been reports of diffuse panbronchiolitis (DPB) occurring in conjunction with these neoplasms as well. Both bronchiectasis and DPB are potentially raised from an aberrant immune response involving lymphocytes [6]. Based on what we know, there have been only three reported cases of concurrent MG and DPB. Notably, MG was diagnosed simultaneously with thymoma in all three cases.

Herein, we present a patient who developed DPB and PTMG sequentially several years after thymectomy.

## Case presentation

**Fig. 1:**
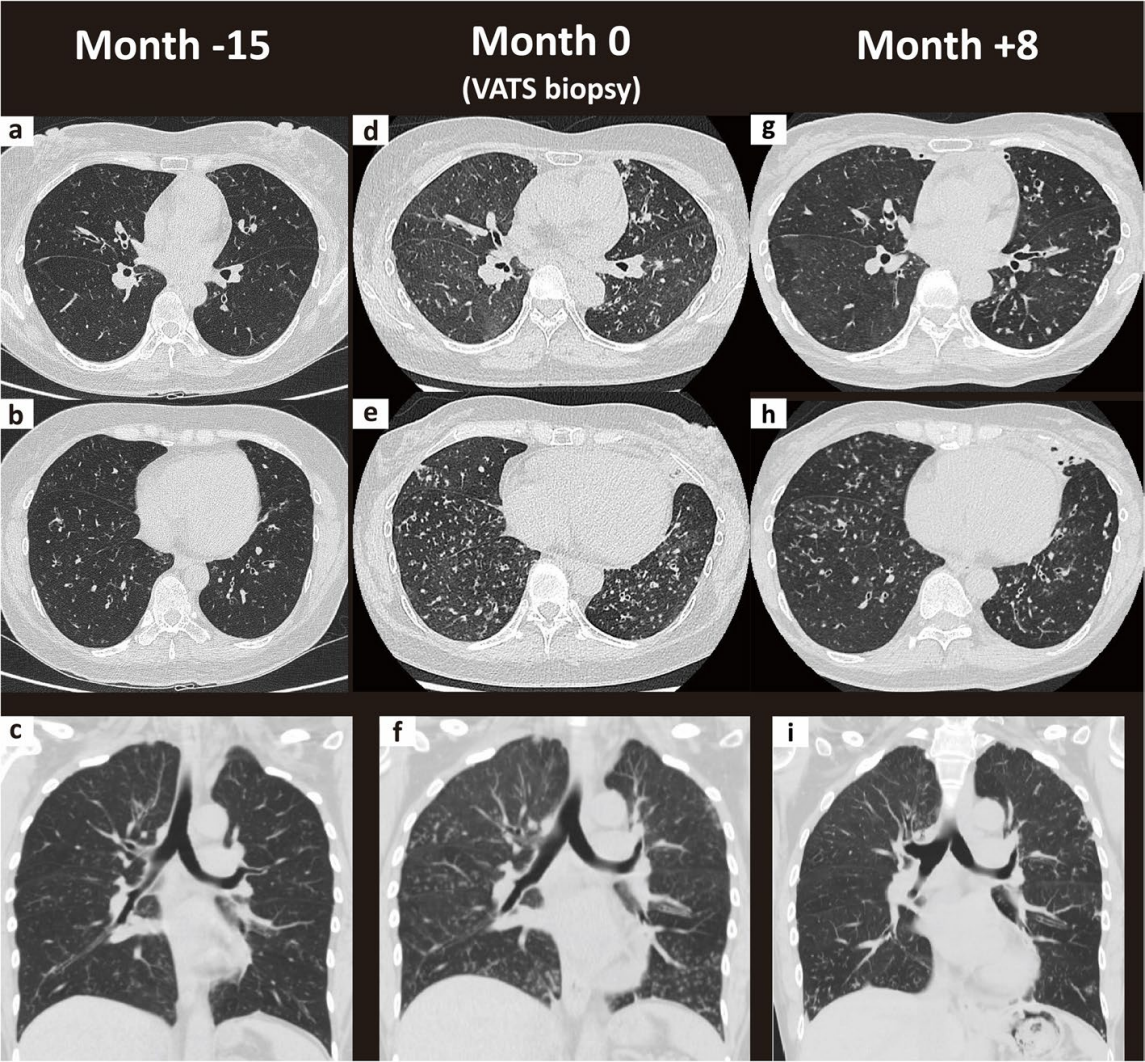
Axial and coronal views of chest computed tomography scan. **a, b, c** At 15 months before this presentation. **d, e, f** At the time of admission for video-assisted thoracoscopic surgery (VATS) biopsy. **g, h, i** At 8 months after DPB diagnosis, in non-exacerbation phase

A 45-year-old Taiwanese woman who had a productive cough for 1.5 years, presented at our hospital in November 2022. The patient also complained of rhinorrhea, anosmia, intermittent ear fullness, shortness of breath, and an 18% weight loss within half a year. She had no fever, chest pain, hemoptysis, or night sweating. She never smoked. Further, her past history was notable for a thymoma, and she had undergone thymectomy with adjuvant radiotherapy 7 years earlier. The follow-up chest CT scans six years after thymectomy revealed increased diffuse bronchitis and bronchiolitis over both lungs **(**Fig. 1a, b, c**)**. She was once referred to the otolaryngologist’s clinic for rhinorrhea, anosmia and ear fullness, where a sinus CT scan and local findings confirmed the diagnosis of chronic pansinusitis and chronic left otitis media.

**Fig. 2:**
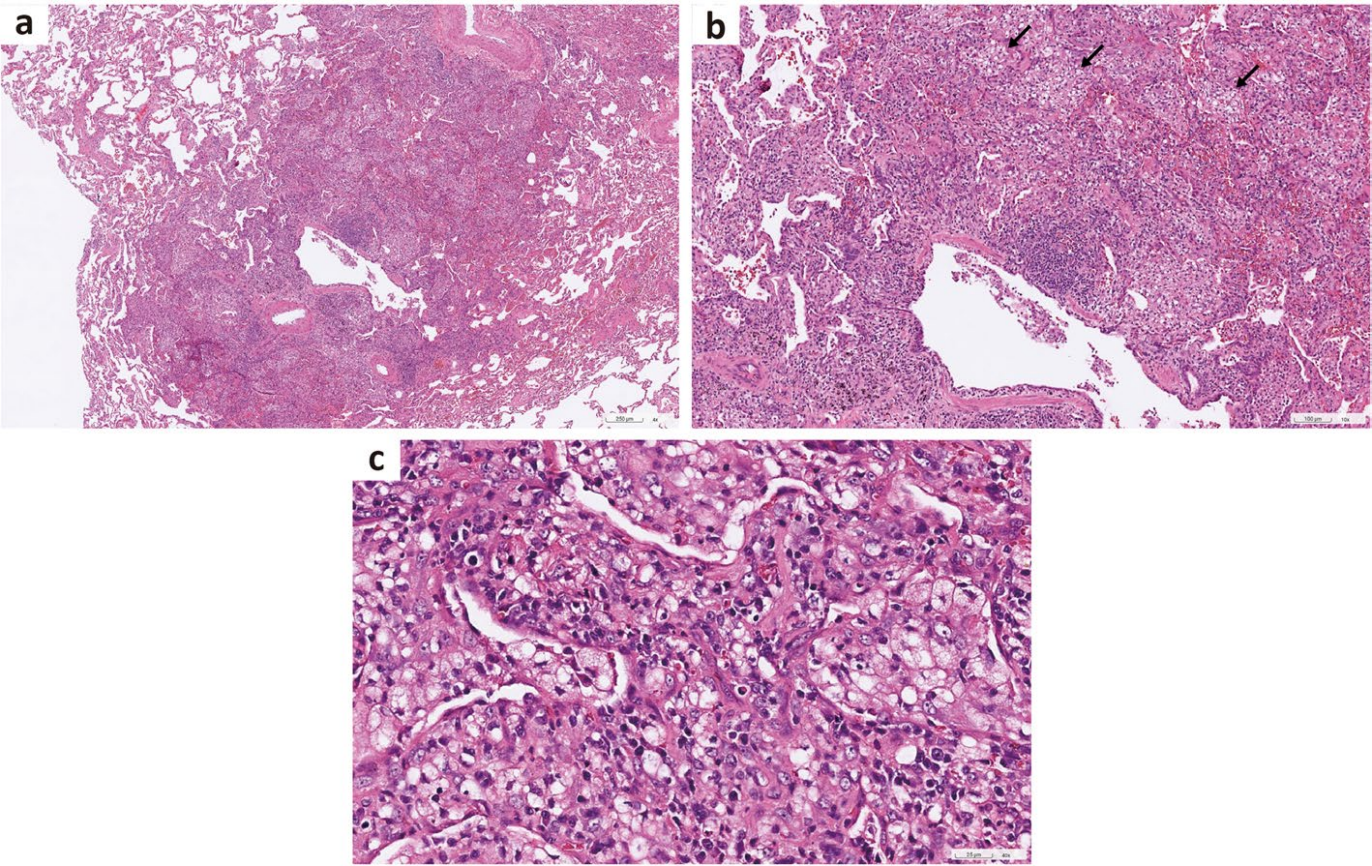
Video-assisted thoracoscopic surgery (VATS) lung biopsy. **a** Bronchiolocentric inflammation involving the bronchioles and respiratory bronchioles. (hematoxylin and eosin stain, × 4). **b** Inflammatory infiltrates including lymphocytes, plasma cells, and foamy histiocytes (arrow) in the alveolar spaces and interstitium. (hematoxylin and eosin stain, × 10). **c** Aggregation of foamy histiocytes in the alveolar spaces and interstitium. (hematoxylin and eosin stain, × 40)

The patient was admitted to the ward for further evaluation of the underlying etiology. Physical examination revealed coarse breathing sounds with inspiratory crackles. Chest CT scan revealed diffuse bronchial wall thickening and mild bronchiectasis with multiple centrilobular nodules, ground-glass nodules, and air trapping **(**Fig. 1d, e, f**)**. The results of the initial and serial spirometry as well as respiratory muscle strength are presented in Table 1. All routine blood biochemistry and autoimmune profiles, including immunoglobulin levels, were within normal limits. Moreover, cultures of the bronchial alveolar lavage did not yield significant growth. To investigate the underlying etiologies, she underwent video-assisted thoracoscopic surgery (VATS) lung biopsy, and the pathological report confirmed the diagnosis of DPB **(**Fig. 2**)**. Long-term azithromycin therapy at a dose of 500 mg/day was initiated. During the event that the patient presented with productive cough with yellowish or greenish sputum, the initial sputum bacterial cultures grew wild-type *Pseudomonas aeruginosa* and intravenous antipseudomonal antibiotics were administered for two weeks, followed by three months of amikacin inhalation therapy aimed at eradicating the pathogen. However, a repeated bacterial sputum culture again identified wild-type *P. aeruginosa*, indicating that the eradication attempt was unsuccessful. Oral levofloxacin therapy, administered at a dosage of 750 mg daily for a 10 to 14-day course, remained partially effective at improving her symptoms during episodes of acute exacerbation in the outpatient setting, despite the bacterial pathogens becoming drug-resistant in her subsequent sputum cultures.

**Table 1:** Diagnostic timeline and serial respiratory function test results

	Month 0 (VATS biopsy)	Month + 3 (MG)	Month + 4	Month + 8	Month + 10
FEV1 (% predicted)	47.7	46.9	66.6	43.4	45.9
FVC (% predicted)	53.1	49.1	68.1	52.5	52.6
FEV1/FVC	0.776	0.824	0.844	0.713	0.752
PImax/PEmax (cmH_2_O)	NA	NA	− 81/ + 107	− 52/ + 50	− 88/ + 92

*FEV1* forced expiratory volume in 1 s, *FVC* forced vital capacity, *%predicted* percentage of the predicted value, *NA* Not available, *PImax* maximal inspiratory pressure, *PEmax* maximal expiratory pressure

**Fig. 3:**
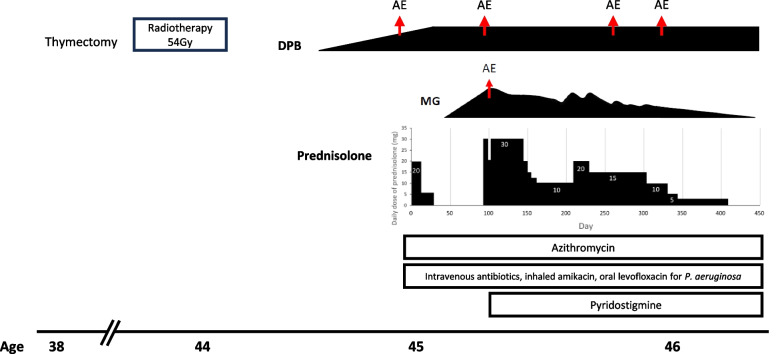
Clinical course (AE: acute exacerbation)

Three months after DPB diagnosis, the patient experienced a gradual onset of symptoms, which included facial weakness, head drop, diplopia, and proximal muscle weakness. Subsequently, she had ptosis with diurnal fluctuations and dysphagia. Arterial blood gas analysis revealed hypercapnia with a partial carbon dioxide pressure of 78.6 mmHg, a bicarbonate level of 39.9 mmol/l, and a pH of 7.32. Noninvasive positive pressure ventilation support (NIPPV) was administered. The pulmonary function test revealed deteriorated FVC (Table 1). Due to the aforementioned symptoms, the acetylcholine receptor antibody test was conducted. Results revealed a high level of acetylcholine receptor antibody at 7.616 nmol/l, which is significantly above the normal range (< 0.2 nmol/l). The repetitive stimulation test also showed a decremental change in the resting and postexercise test results. These findings indicated an abnormality at the neuromuscular junction. Thus, the patient was diagnosed with MG (Fig. 3).

Treatment with prednisolone at a daily dose of 0.5 mg/kg and pyridostigmine bromide at a dose of 60 mg four times a day was initiated. Thereafter, ptosis resolved and proximal weakness improved. Consequently, the patient was gradually weaned off NIPPV support. During stable periods without acute exacerbations of DPB, chest CT scan revealed improvement of bronchiolitis but persistent bronchiectasis (Fig. 1g, h, i). Her exertional dyspnea persisted, requiring 1 to 2 L per minute of oxygen during short walks. She spent most of her daily activities indoors without going out or exercising. Currently, oral corticosteroids have been gradually tapered off over ten months to reduce infection risks. She receives long-term maintenance therapies with pyridostigmine, azithromycin and inhaled amikacin. Nonetheless, due to colonization by quinolone-resistant *P. aeruginosa*, hospitalization for intravenous antibiotic treatment may occasionally be necessary if acute exacerbations occur with severe dyspnea and desaturation.

## Discussion and Conclusions

In this report, we describe a case who develops DPB and PTMG sequentially several years after thymectomy for thymoma. We presented a complex clinical course and diagnostic challenge, and under careful clinical assessment, the patient was diagnosed and treated correctly and appropriately in a timely manner.

DPB typically manifests between the ages of 20 and 50, with a higher prevalence in males compared to females. This disease primarily affects individuals in Asian populations. Certain HLA types have been found to be associated with the disease [7]. Greater than 80% of patients with DPB have a history of chronic paranasal sinusitis, or they continuously present with the condition [7]. In the current case, the patient was initially diagnosed with sinusitis, which exhibited features consistent with those described in the literature. While macrolide therapy improves survival in DPB patients, those with coexisting bronchiectasis are at increased risk of *P. aeruginosa* infections, which leads to a poorer prognosis for maintenance macrolide therapy [8]. Lung transplantation might be a feasible long-term treatment solution for progressively worsening DPB. In a previous case series [9], five DPB patients who deteriorated despite macrolide therapy all showed colonization with *P. aeruginosa*. These five patients eventually required bilateral lung transplantation and remained alive with a median survival time of 4.9 years after transplantation, without recurrence of DPB.

To assess the association between thymoma and DPB, we evaluated the data of 16 patients from reports published in PubMed, Medline, and Web of Science, using the terms “diffuse panbronchiolitis” and “thymoma” or “thymic carcinoma” (Table 2). Eight patients concomitantly experienced thymoma and DPB, and the others developed DPB at different time points after thymectomy.

**Table 2:** Reported cases of thymoma complicated with diffuse panbronchiolitis

Age/sex	Country	Time to DPB diagnosis	MG	Time to MG diagnosis	Other complications of thymoma	Treatment	Outcome	Reference
69/M	Japan	2.5 years after thymectomy	-	NA	Good syndrome	Immunoglobulin replacement	Death	[10]
58/F	Japan	Simultaneous	-	NA	Nil	Macrolide	Death	[11]
58/M	Japan	Simultaneous	-	NA	Good syndrome	Immunoglobulin replacement	NA	[12]
15/M	China	Simultaneous	-	NA	Nil	NA	NA	[13]
54/M	China	Simultaneous	-	NA	Nil	NA	NA	[13]
22/F	China	Simultaneous	-	NA	Nil	Macrolide	Improvement	[14]
54/M	China	Simultaneous	-	NA	Nil	Macrolide	Improvement	[14]
65/F	Japan	Simultaneous	-	NA	Good syndrome, pure red cell aplasia	Macrolide	Improvement	[15]
41/F	China	1 year after thymectomy	-	NA	Nil	Macrolide	Improvement	[16]
70/M	China	5 years after thymectomy	-	NA	Nil	Macrolide	Improvement	[17]
50/M	India	6 months after thymectomy	-	NA	Nil	Macrolide	Improvement	[18]
67/F	China	1 year after thymectomy	-	NA	Good syndrome	Macrolide	Improvement	[19]
69/M	Caucasian	1 year after thymectomy	-	NA	Nil	Macrolide	Improvement	[20]
27/M	China	Simultaneous	+	Simultaneous	Nil	Macrolide	Improvement	[21]
45/M	Japan	9 moths after thymectomy	+	Simultaneous	Good syndrome, pure red cell aplasia	Corticosteroid, cyclosporine, Macrolide	Improvement	[22]
58/M	Japan	12 years after thymectomy	+	Simultaneous	Alopecia, dysgeusia, myositis	Macrolide, corticosteroid	Death due to DPB progression and fatal respiratory infection	[23]

Previous studies have presented the possible etiologies of bronchiectasis in thymic neoplasms [2, 3]. Immune system irregularities related to thymic neoplasms might be the underlying etiology for DPB development. That is, abnormal immune responses could affect the bronchi and respiratory bronchioles. The other two possible etiologies were recurrent respiratory tract infection in patients with Good syndrome and expectoration difficulties in patients with MG.

Of the 16 patients, 3 were diagnosed with MG and DPB. One patient developed concomitant MG and DPB upon thymoma diagnosis. The other two patients developed DPB several years after thymectomy. In the case report of Ogoshi et al., the patient presented with recurrent lower respiratory infections 9 months after the thymectomy where neurological abnormalities were not observed. Considering the patient's negative reaction to cold agglutinin, low globulin levels, and reduced B lymphocytes in the blood, there might be the possibility of Good syndrome with bronchopulmonary lesions resembling DPB. Although the B lymphocyte count in the peripheral blood decreased, B lymphocytes surrounded the terminal bronchioles in this case. This phenomenon could be a contributing factor to DPB development in patients with Good syndrome [22]. In this case, DPB developed after MG was effectively treated. In our case, DPB developed before symptoms of MG were present. Expectoration difficulty may have had minimal contribution to the development of DPB. Consequently, immune dysregulation can be the main underlying factor for DPB in patients with a history of thymic neoplasms.

In the previous cases, patients diagnosed with thymoma and DPB (Table 2) commonly received macrolide therapy. Two of five patients with Good syndrome received immunoglobulin replacement. In terms of outcomes, 3 of 16 patients eventually died. One patient died of progressive DPB and severe respiratory infection.

The clinical features of PTMG were similar to those of prethymectomy MG [24], and the long-term overall survival was not significantly affected by MG development after thymectomy [25]. Pyridostigmine is the preferred choice for patients with symptoms and corticosteroids and azathioprine for those who do not sufficiently respond to symptomatic therapy [4].

However, the therapeutic approach for both MG and DPB presents complex challenges. Macrolide therapy is essential for DPB, and antibiotic quinolones play a key role in treating respiratory tract infections caused by *P. aeruginosa* in the outpatient setting. However, the use of macrolide and quinolone antibiotics both comes with the potential of worsening MG. Aminoglycosides can also exacerbate MG. Contrary to expectations, our case showed that inhaled amikacin might be a safe therapeutic approach for patients with MG who presented with *P. aeruginosa*-associated lung infection. The treatment of steroid-dependent patients is another challenge. A reduced steroid dose worsens MG symptoms. Meanwhile, an increased dose may exacerbate DPB.

To the best of our knowledge, this is the first case report on the sequential development of DPB followed by PTMG. Our case sheds light on the persistent state of immune dysregulation in patients with thymoma, even after thymectomy, with various temporal onsets. DPB diagnosis should be considered in patients exhibiting respiratory symptoms, recurrent respiratory infections, and radiological indications such as diffuse nodules or bronchiectasis. Further studies should be performed to investigate strategies for preventing or reducing immune dysregulation after thymectomy.

## Data Availability

The data and material that support this case report are available from the corresponding author on reasonable request.

## Abbreviations

MG: Myasthenia gravis
PTMG: Post-thymectomy myasthenia gravis
DPB: Diffuse panbronchiolitis
CT: Computed tomography
